# *par-1*, Atypical *pkc*, and PP2A/B55 *sur-6* Are Implicated in the Regulation of Exocyst-Mediated Membrane Trafficking in *Caenorhabditis elegans*

**DOI:** 10.1534/g3.113.006718

**Published:** 2013-11-05

**Authors:** Yaming Jiu, Kiran Hasygar, Lois Tang, Yanbo Liu, Carina I. Holmberg, Thomas R. Bürglin, Ville Hietakangas, Jussi Jäntti

**Affiliations:** * Institute of Biotechnology, Research Program in Cell and Molecular Biology, 00014 University of Helsinki, Helsinki, Finland; † Department of Biosciences, University of Helsinki, Helsinki, Finland; ‡ Department of Biosciences and Nutrition, and Center for Biosciences, Karolinska Institutet, Novum, SE 141 83, Huddinge, Sweden; § Research Programs Unit, Translational Cancer Biology, and Institute of Biomedicine, 00014 University of Helsinki, Helsinki, Finland; ** VTT Technical Research Centre of Finland, Espoo, 02044 VTT, Finland

**Keywords:** *Caenorhabditis elegans*, exocyst, PP2A, par-1, pkc-3

## Abstract

The exocyst is a conserved protein complex that is involved in tethering secretory vesicles to the plasma membrane and regulating cell polarity. Despite a large body of work, little is known how exocyst function is controlled. To identify regulators for exocyst function, we performed a targeted RNA interference (RNAi) screen in *Caenorhabditis elegans* to uncover kinases and phosphatases that genetically interact with the exocyst. We identified seven kinase and seven phosphatase genes that display enhanced phenotypes when combined with hypomorphic alleles of *exoc-7* (*exo70*), *exoc-8* (*exo84*), or an *exoc-7;exoc-8* double mutant. We show that in line with its reported role in exocytotic membrane trafficking, a defective *exoc-8* caused accumulation of exocytotic soluble NSF attachment protein receptor (SNARE) proteins in both intestinal and neuronal cells in *C. elegans*. Down-regulation of the phosphatase protein phosphatase 2A (PP2A) phosphatase regulatory subunit *sur-6/*B55 gene resulted in accumulation of exocytic SNARE proteins SNB-1 and SNAP-29 in wild-type and in *exoc-8* mutant animals. In contrast, RNAi of the kinase *par-1* caused reduced intracellular green fluorescent protein signal for the same proteins. Double RNAi experiments for *par-1*, *pkc-3*, and *sur-6/*B55 in *C. elegans* suggest a possible cooperation and involvement in postembryo lethality, developmental timing, as well as SNARE protein trafficking. Functional analysis of the homologous kinases and phosphatases in *Drosophila* median neurosecretory cells showed that atypical protein kinase C kinase and phosphatase PP2A regulate exocyst-dependent, insulin-like peptide secretion. Collectively, these results characterize kinases and phosphatases implicated in the regulation of exocyst function, and suggest the possibility for interplay between the *par-1* and *pkc-3* kinases and the PP2A phosphatase regulatory subunit *sur-6* in this process.

The exocyst is a functionally and structurally conserved multiprotein complex that is essential for the regulation of cell polarity in eukaryotic cells. It is involved in targeting and tethering of transport vesicles to the plasma membrane (Hsu *et al.* 2004; Munson and Novick 2006; He and Guo 2009; St Johnston and Ahringer 2010), and it is composed of eight subunits: Sec3 (human EXOC1), Sec5 (h EXOC2), Sec6 (h EXOC3), Sec8 (h EXOC4), Sec10 (h EXOC5), Sec15 (h EXOC6), Exo70 (h EXOC7, *Caenorhabditis elegans exoc-7*), and Exo84 (h EXOC8, *C. elegans exoc-8*) (TerBush *et al.* 1996) (www.genenames.org). Although *C. elegans* is a widely used model system in developmental biology, thus far the exocyst is poorly characterized in the worm. It has been reported that exocyst complex genes affect localization of apical actin in mutants of *wts-1*, the *C. elegans* Lats kinase homolog (Kang *et al.* 2009). WTS-1/Lats plays important roles for the structural integrity of the apical membrane in intestinal cells. The small Ras-like GTPase *rap-1* has been shown to act in concert with the RAL-1/exocyst pathway in mediating hypodermal cell movement and elongation during embryonic development in *C. elegans* (Frische *et al.* 2007). In addition, silencing of *ral-1*, *exoc-8*, *sur-6*, and *vhp-1* caused synthetic lethality in *rap*-mutant animals (Frische *et al.* 2007). Recently, we showed that hypomorphic mutant alleles of *exoc-7* and *exoc-8* cooperate with RAB-10 GTPase in endocytic membrane trafficking in intestinal epithelial cells in *C. elegans* (Jiu *et al.* 2012).

Studies that use different model systems indicate an important interplay between the exocyst and small GTPases, which regulate many steps in intracellular membrane traffic (Mizuno-Yamasaki *et al.* 2012). Evidence is mounting that the regulation of small GTPase interactions with exocyst components is phosphorylation dependent. For example, in yeast phosphorylation of the RAB GTPase Sec4p is implicated in regulating the interaction of Sec4p with the exocyst subunit Sec15p (Heger *et al.* 2011). The mammalian exocyst interacts with atypical protein kinase C (aPKC) in regulating cell migration, suggesting that members of this kinase family could fine-tune exocyst function through phosphorylation of exocyst subunits (Rosse *et al.* 2009). Furthermore, the interaction between RalA GTPase and the exocyst subunit Sec5p was negatively regulated by PKC-dependent phosphorylation of Sec5 in mammalian cells (Chen *et al.* 2011a). Mutations in Sec5 that abolished its phosphorylation-dephosphorylation capacity perturbed insulin-dependent GLUT4 exocytosis in adipocytes (Chen *et al.* 2011a). Furthermore, it has been shown that insulin-stimulated GLUT4 storage vesicle exocytosis is regulated by exocyst complex function and involves the Ser/Thr kinase AKT (Stockli *et al.* 2011).

Analysis of phosphopeptides in several model systems has identified potential *in vivo* phosphorylation sites in the exocyst subunits (*e.g.*, http://www.phosphosite.org/homeAction.do, http://www.phosida.com/). However, the connections between phosphorylation-mediated signaling and exocyst function have not been systematically addressed. The *C. elegans* genome contains more than 400 kinases and almost 200 phosphatases, and most of them have mammalian orthologs. In this study, an RNA interference (RNAi) screen was performed to identify kinases and phosphatases that are functionally linked to the exocyst components EXOC-7 and EXOC-8. The screen identified seven kinases and seven phosphatases. The results suggest that *par-1* and *pkc-3* may directly or indirectly cooperate with *sur-6*, a serine/threonine phosphatase protein phosphatase 2A (PP2A) subunit B, in the regulation of postembryonic growth, embryonic development, and intracellular transport of soluble NSF attachment protein receptor (SNARE) complex subunits. Finally, we tested homologs for the identified genes in another model system, *i.e.*, the secretion of *Drosophila* insulin-like peptides that is also exocyst dependent. This analysis showed an essential role for aPKC and PP2A in this process.

## Materials and Methods

### *C. elegans* strains

Strains used in this study were: N2(wild type), rrf-3(pk1426), exoc-7(ok2006), exoc-8(ok2523), unc-18(e81), B0252.1(gk1114), RT1585 pwIs603[Pvha-6::gfp::snb-1], RT1944 pwIs695[Pvha-6::gfp::snap-29], RT1931 pwIs686[Pvha-6::gfp::syn-4], KM246 Is[Pida-1::ida-1::gfp], KP3819 nuIs152[Pttx-3::mrfp+Punc-129::gfp::snb-1], and KP5929 nuIs163[Pmyo-2::gfp+Punc-129::snn-1::venus]. All strains were maintained utilizing standard methods (Brenner 1974). Other strains with different mutant backgrounds were made by crossing: exoc-7(ok2006);exoc-8(ok2523), exoc-7(ok2006);rrf-3(pk1426), exoc-8(ok2523);rrf-3(pk1426), exoc-7(ok2006);exoc-8(ok2523);rrf-3(pk1426), exoc-8(ok2523);pwIs603[Pvha-6::gfp::snb-1], unc-18(e81);pwIs603[Pvha-6::gfp::snb-1], exoc-8(ok2523);pwIs695[Pvha-6::gfp::snap-29], exoc-8(ok2523);pwIs686[Pvha-6::gfp::syn-4], exoc-8(ok2523);Is[Pida-1::ida-1::gfp], exoc-8(ok2523);nuIs152[Pttx-3::mrfp+Punc-129::gfp::snb-1], and exoc-8(ok2523);nuIs163[Pmyo-2::gfp+Punc-129::snn-1::venus].

### RNA interference

The RNAi screen was performed by feeding bacterial clones (J. Ahringer RNAi library; Geneservice) on six-well nematode growth media plates containing 1 mM isopropylthiogalactoside to *rrf-3*, *rrf-3;exoc-7*, *rrf-3;exoc-8* and *rrf-3;exoc-7;exoc-8* mutant animals that had been synchronized at the L1 stage (Fraser *et al.* 2000). The animals were allowed to grow for 3 d before observing the phenotype. In total, we tested 246 kinase genes and 167 phosphatase genes. All kinase and phosphatase RNAi clones were tested in duplicates, and the candidate genes were confirmed in three additional independent experiments and subsequently retested in the N2 background with at least two repetitions. To verify the identities of the identified candidate genes, the RNAi clones in the library were verified by sequencing. To achieve comparable RNAi dosages in the double RNAi experiments, the corresponding single RNAi controls were carried out by mixing equal amounts of the target gene expressing RNAi *Escherichia coli* with *E. coli* that contains only the empty vector (L440).

### Confocal microscopy

All static microscope images were acquired using a Leica TCS SP5 Laser scanning confocal microscopy with 20× glycerol objective. Confocal settings used for image capture were held constant for the same marker strains in the experiments. Images were quantified and analyzed using ImageJ software (National Institutes of Health). The worm fluorescence imaging and quantification were done as previously described (Yu *et al.* 2011). The average pixel intensity in wild type worms was set to an arbitrary fluorescence unit (A.U.) of 1.0 to enable comparison with other strains.

### Western blot assay

For analysis of green fluorescent protein (GFP) levels, worms from 9-cm plates were harvested by centrifugation at about 360g for 3 min. After they were washed with M9 buffer three times, worms were directly boiled for 10 min in 300 μL of 2% sodium dodecyl sulfate buffer containing protease inhibitors. Each sample was centrifuged for 5 min at 13,000 rpm and the protein concentration was determined with a protein assay kit (Thermo Scientific). Identical amounts of total protein was subjected to sodium dodecyl sulfate-polyacrylamide gel electrophoresis and transferred to nitrocellulose membrane (D106089, BIO-RAD) following standard procedures. The GFP-tagged proteins were detected with anti-GFP antibody (98028, BD). α-tubulin was used for normalization.

### 4D embryo recording

4D time lapse microscopy was performed as previously described (Hench *et al.* 2009), with the modification that a 20× objective and a light-emitting diode light source was used for DIC. To collect data for the time interval that the embryos of different strain backgrounds needed to develop from the 4-cell stage embryo to the 28-cell stage embryo, the recordings always contain at least one embryo that is before the 4-cell stage. The recordings were subsequently viewed and analyzed with the use of Endrov (Henriksson *et al.* 2013, www.endrov.net).

### RNA isolation and qRT-PCR

Animals subjected to RNAi were collected and washed 3 times with M9. After removing the supernatant, worm pellets were stored at −80° until RNA isolation. The total RNA was extracted from approximately 1000 animals for each treatment using the Total RNA Isolation kit (Macherey-Nagel, Düren, Germany), and first-strand cDNA was synthesized using the Maxima First-Strand Synthesis Kit for RT-qPCR (ThermoScientific). SYBR green real-time Quantitative PCR (qRT-PCR) was carried out using the LightCycler 480 Real-Time PCR System (Roche). In each qRT-PCR assay, we used three biological replicates, and experiments were repeated two times. α-tubulin was used for normalization. Primer sequences are available in Supporting Information, Table S1.

### Fly stocks

The *Drosophila* insulin-like peptide 2 (dILP2)-Gal4, UAS-GFP (Rulifson *et al.* 2002) driver line was used for all experiments with *Drosophila*. The W^1118^ line crossed to dILP2-Gal4, UAS-GFP was used as a control. RNAi lines were obtained from the Vienna Drosophila RNAi Center (RNAi_aPKC, RNAi_Pp2A, RNAi_Sec3, RNAi_Sec6, RNAi_Sec8, RNAi_Sec10, RNAi_Sec15, and RNAi_Exo84) and the Bloomington *Drosophila* Stock Center (RNAi _aPKC). For all experiments, flies were allowed to lay eggs at 25° and L1 larvae were transferred to 29°. Starvation: Nonwandering L3 larvae were transferred to agar plates containing phosphate-buffered saline (PBS) +1% sucrose for 20 hr at 29°.

### Drosophila weight measurements

UAS-RNAi flies were crossed to dILP2-Gal4, UAS-GFP flies and allowed to lay eggs at 25° for 6 hr. L1 larvae were collected (80 per vial) 24 hr after egg deposition and grown at 29°. Four days after adult emergence, male flies with both dILP2-Gal4 and RNAi or RNAi-only (control) were weighed in groups (≥10 flies/group, N ≥ 3), using a Mettler AF 200 fine balance. The weight of the insulin-producing cell (IPC)-specific knockdown flies was normalized by the weight of control flies from the same vial.

### *Drosophila* immunostaining

Brains were dissected from wandering 3rd instar larvae, fixed in 4% formaldehyde-PBS for 30 min at room temperature, and washed in PBT (0.3% Triton X 100 in PBS). After blocking in 5% bovine serum albumin in PBT for 2 hr at room temperature, primary antibodies were incubated at 4° o/n. Primary antibodies were washed (3× 15 min) with PBT, and secondary antibodies were incubated for 2 hr at room temperature. After washes, brains were mounted in Vectashield Mounting Medium (Vector Laboratories, Inc., Burlingame, VT). Fluorescence images were taken using a Leica TCS SP5 MP SMD FLIM confocal laser scanning microscope. Antibodies used: rat anti-dILP2 (Geminard *et al.* 2009), anti-rat Alexa fluor 633 (1:400, Invitrogen). Twelve-bit confocal images of each IPC cluster were acquired using the same scan and laser power settings. Total signal from each cluster was quantified using Image J software, and results were analyzed in Microsoft Excel.

### Data analysis

Data analysis was conducted using IGOR Pro (Wavemetrics), EXCEL (Microsoft), or SigmaPlot software. Averaged results were presented as the mean value ± SEM. Statistical significance was evaluated either using Student’s *t*-test or Mann-Whitney test. Asterisks denote statistical significance as compared to controls, with a *P* value less than 0.05 (*), 0.01 (**) and 0.001 (***).

### Results

### A synthetic lethality RNAi screen for kinases and phosphatases that enhance *exoc-7* and *exoc-8* mutant phenotypes

**Figure 1:**
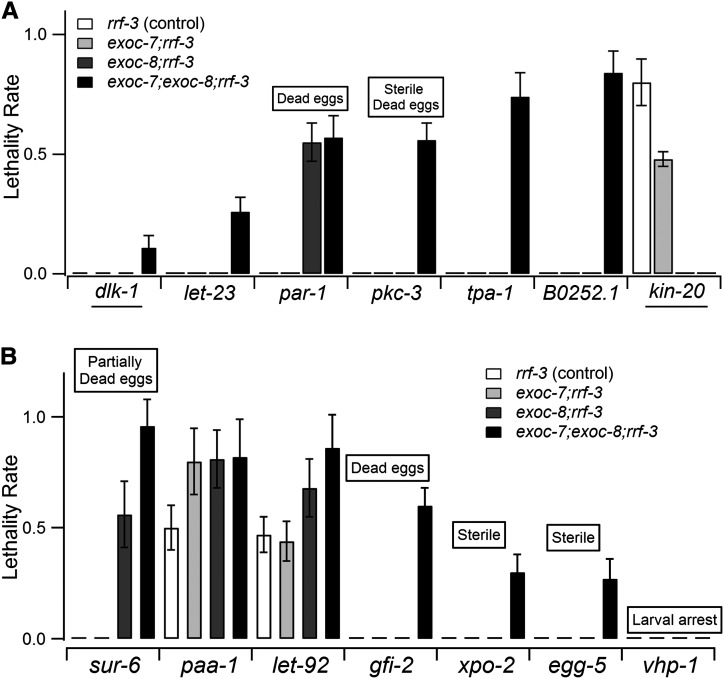
RNAi screen for kinases and phosphatases reveals novel genetic interactors for *exoc-7* and *exoc-8*. The histogram shows synthetic lethality rates of the candidates identified in the kinase screen (A) and the phosphatase screen (B). Boxes above the columns list the most prominent phenotypes caused by silencing of the corresponding genes in the *rrf-3* background. Underlining indicates genes where the enhanced phenotype was not phenocopied in the wild-type N2 background.

*exoc-7(ok2006)* and *exoc-8(ok2523)* are hypomorphic mutants that result in compromised neuronal function and defective membrane trafficking in *C. elegans* intestine epithelial cells (Jiu *et al.* 2012). These mutants are the only known weak alleles of genes encoding exocyst complex subunits in *C. elegans*. To address, whether phosphoregulation contributes to exocyst function in *C. elegans*, a synthetic lethality RNAi screen was performed for *C. elegans* kinases and phosphatases in *exoc-7(ok2006)* and *exoc-8(ok2523)* single mutant animals, as well as in *exoc-7(ok2006);exoc-8(ok2523)* double-mutant animals. The screen was performed by transferring synchronized L1 worms to RNAi plates and observing the phenotypes 3 d thereafter. Under these conditions the non-RNAi control animals grew to adults (Jiu *et al.* 2012). Six of 246 kinases and seven of 165 phosphatases (available in Ahringer RNAi library) were identified that cause synthetic lethality when combined with *exoc-7*, *exoc-8*, or *exoc-7;exoc-8* mutants in the RNAi-sensitive *rrf-3* background (Figure 1). Surprisingly, the lethality induced by RNAi of *kin-20*, the seventh kinase hit, was partially suppressed in *exoc-7*, and completely suppressed in *exoc-8* and *exoc-7;exoc-8* animals. *par-1*, *paa-1*, and *let-92* RNAi caused synthetic lethality of variable extent [40% (*let-92*) to 80% (*paa-1*)] with the single *exoc-7* or *exoc-8* mutations. However, typically, the most severe phenotypes were observed when RNAi was performed in *exoc-7;exoc-8* double mutants (Figure 1). With the exception of *dlk-1*, and *kin-20*, which were only identified in the *rrf-3* background, the other hits could also be verified in the wild-type N2 background (data not shown). All of the genes identified are highly conserved in metazoans (Table 1).

**Table 1:** General description of the genes identified in the screens

RNAi Clone	Gene	Mammalian Homolog	Description
F33E3.2	*dlk-1*	Dual leucine zipper-bearing kinases (DLKs)	A mitogen-activated protein kinase kinase kinase (MAPKKK)
ZK1067.1	*let-23*	Epidermal Growth Factor Receptor (EGFR)	A receptor tyrosine kinase, genetically upstream of the let-60/RAS pathway
H39E23.1	*par-1*	MARK2	A serine-threonine kinase; essential for early embryonic polarity, asymmetrically localized to the posterior of late one-cell embryos
F09E5.1	*pkc-3*	αPKC	An atypical protein kinase; essential for establishing anterior-posterior polarity, localizes to the anterior periphery of the one-cell embryo
B0545.1a	*tpa-1*	PKCδ	Protein kinase C, plays a role in nicotine-induced adaptation and *gpa-12*/G protein-mediated signaling
B0252.1	*B0252.1*	FGFR2	A putative homolog of fibroblast growth factor receptor homolog 2, tyrosine kinase
F46F2.2	*kin-20*	casein kinase 1ε	Kinase, a homolog of the *Drosophila* circadian clock genes doubletime
F26E4.1	*sur-6*	PPP2R2A	A regulatory (B) subunit of serine/threonine protein phosphatase 2A (PP2A-B), B55 family. PR55/B, suppresses *let-60 ras(gf)* Muv, enhances Vul and rod-like lethal phenotypes of *lin-45* hypomorphs
F48E8.5	*paa-1*	PR65	PR65, structural subunit A of protein phosphatase 2A (PP2A). Interacts with SMG-5; required for embryonic viability, fertility, and cuticular integrity.
F38H4.9	*let-92*	PP2AC	PP2AC, the catalytic subunit C of protein phosphatase 2A (PP2A). Required for embryogenesis, larval development, Ras-mediated vulval development, and axonal guidance
K02A11.1	*gfi-2*	PPP1R16A, Protein phosphatase 1 regulatory subunit 16A	GEI-4 interacting protein
Y48G1A.5	*xpo-2*	Exportin-2	Essential for embryogenesis and required for normal pronuclear envelope dynamics and vulval morphogenesis
R12E2.10	*egg-5*	Tyrosine-protein phosphatase nonreceptor type 7	A pseudotyrosine phosphatase; required for the egg-to-oocyte transition and embryonic development
F08B1.1	*vhp-1*	MKP7	Required for regulation of the KGB-1/JNK-like MAPK-mediated stress response pathway

RNAi, RNA interference.

*par-1* and *pkc-3* belong to the major cell polarization regulator PAR protein family (Table 1). *par-1* encodes a serine/threonine kinase, which is essential for establishing early embryonic polarity. PAR-1 is present in the cytoplasm and in the cell cortex and is asymmetrically localized to the posterior cortex of late one-cell stage embryos (Guo and Kemphues 1995). As previously reported, *par-1* RNAi caused dead F1 eggs (Figure 1) (Guo and Kemphues 1995). *pkc-3* encodes an atypical protein kinase, which localizes to the anterior periphery of the one-cell embryo and plays an essential role in establishing anteroposterior polarity (Izumi *et al.* 1998; Tabuse *et al.* 1998). Knockdown of *pkc-3* caused sterility and F1 egg lethality, whereas the maternal P0 worms showed no obvious defects. The currently uncharacterized open reading frame *B0252.1* encodes a homolog of *S. cerevisiae* Cla4p, a Cdc42p activated PAK-family kinase required for cytokinesis but also other processes (Gulli *et al.* 2000; Versele and Thorner 2004). The attempts to combine the viable *B0252.1(gk1114)* allele with *exoc-7;exoc-8* mutations did not yield viable homozygous animals. This genetic interaction supports the existence of a functional link between *B0252.1* and the exocyst complex in *C. elegans*.

In the RNAi screen targeting phosphatases, *sur-6*, *paa-1*, and *let-92* displayed the strongest genetic interactions with the examined exocyst subunits. These genes encode components of the PP2A complex (Table 1). PP2A is a major serine/threonine phosphatase that is abundantly and ubiquitously expressed and has been highly conserved during the evolution of eukaryotes. PP2A plays an important role in regulating multiple signal transduction pathways, including cell-cycle regulation, cell growth and development, cytoskeleton dynamics, and cell motility (Versele *et al.* 2004). PP2A is composed of three protein subunits, A, B, and C, and the core complex of PP2A is composed of tightly associated A and C subunits. The B subunit determines the substrate specificity as well as the spatial and temporal functions of PP2A (Gulli *et al.* 2000; Cheung *et al.* 2004). In *C. elegans*, *paa-1* and *let-92* are the only genes encoding the structural A subunit and the catalytic C subunit, respectively. The B-subunit variants are encoded by seven genes belonging to three families (B55, B56, and B72) in *C. elegans*, and the *sur-6* gene identified in the screen is the only representative of the B55 family.

RNAi of *egg-5* revealed a low percentage (27%) of synthetic lethality with *exoc-7;exoc-8* double-mutant animals. *egg-4*, which encodes a closely related pseudotyrosine phosphatase (99% similarity with *egg-5*), did not show any synthetic lethality with these exocyst mutants. However, *egg-4(RNAi)* worms were sick in the *exoc-7;exoc-8* double-mutant background.

### RNAi phenotypes suggest genetic interactions for *par-1*, *pkc-3*, and *sur-6* in the *exoc-8* mutant background

**Figure 2:**
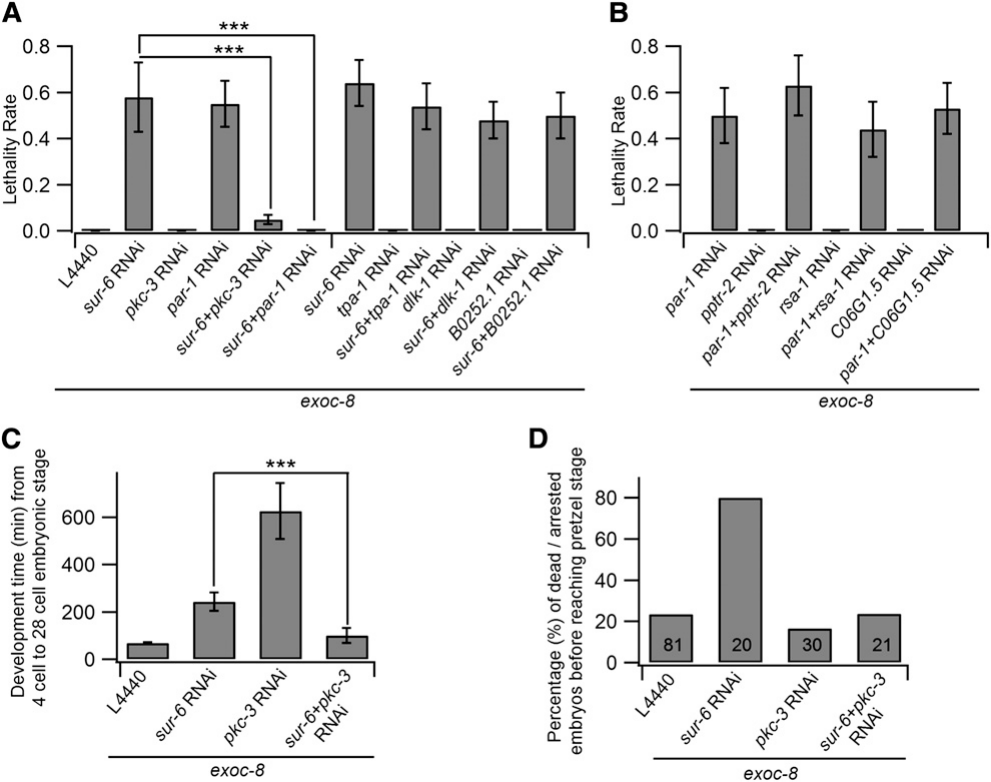
Analysis of the genetic interactions between *par-1*, *pkc-3*, and *sur-6* in an *exoc-8* mutant background. (A) *par-1(RNAi)* and *pkc-3(RNAi)* suppress the growth defects induced by PP2A *sur-6*/*B55(RNAi)* in *exoc-8* mutant animals. In contrast, no suppression is observed in double RNAi experiments with other kinases identified in the screen (*sur-6(RNAi);tpa-1(RNAi)*, *sur-6(RNAi);dlk-1(RNAi)*, or *sur-6(RNAi);B0252.1(RNAi)*). The histogram shows the quantification of the lethality rate from four independent experiments. (B) Analysis of the phosphatase subunit specificity of the *par-1(RNAi)* phenotype suppression. The combination of PP2A *pptr-2(RNAi)*, *rsa-1(RNAi)*, or C06G1.5*(RNAi)* with *par-1(RNAi)* revealed no suppression of the *par-1(RNAi)* induced lethality in the *exoc-8* mutant background. (C) *pkc-3(RNAi)* suppresses the embryonic development defects induced by *sur-6(RNAi)* in *exoc-8* mutant animals. The time (minutes) needed for the embryos to develop from 4-cell stage to 28-cell stage. (D) The percentage (%) of dead/arrested embryos before reaching the pretzel stage. Numbers in the columns indicate the number of embryos that were quantitated. Asterisks denote statistical significance as compared with controls, with a *P* value less than 0.001 (***).

To explore in more detail the functional interactions of the identified kinase and phosphatase genes, we examined the effects of combined RNAi treatments. We observed that the synthetic lethal phenotype of *sur-6(RNAi)* was suppressed by *par-1(RNAi)* or *pkc-3(RNAi)* (Figure 2A). In *exoc-8* mutant animals, the knock-down of either *sur-6* or *par-1* caused approximately 60% lethality (Figure 1 and Figure 2A). However, when both *sur-6* and *par-1* were simultaneously silenced in *exoc-8(ok2523)* the lethality was abolished. Similarly, combined knock-down of *sur-6* and *pkc-3* in *exoc-8(ok2523)* resulted in nearly healthy worms (Figure 2A). To test whether a combination of any of the other identified kinases would result in similar suppression phenotypes in *sur-6(RNAi);exoc-8(ok2523)* animals, combined RNAi was performed with *tpa-1*, *dlk-1*, and *B0252.1*, respectively. In these animals no suppression was observed, although strong growth defects were evident, similar to those observed for *sur-6* single RNAi (Figure 2A).

There are seven genes of the PP2A regulatory B subunit family in *C. elegans*, which are classified into three families, *i.e.*, B55 (*sur-6*), B56 (*pptr-1*, *pptr-2*), and B72 (*rsa-1*, *F47B8.3*, *C06G1.5*, *T22D1.5*), respectively. Four of them, *sur-6*/B55, *pptr-2*/B56, *rsa-1*/B72, and *C06G1.5*/B72 were included in the screen. Except for *sur-6*, knocking down any of the other three genes resulted in no obvious defects in *exoc-8* mutant animals (Figure 2B). RT-PCR analysis showed high efficiency (70–96%) of RNAi silencing for the two kinases *pkc-3* and *par-1*, as well as the phosphatases *sur-6*, *pptr-2* and *rsa-1* (Figure S1). When combined with *par-1* RNAi, no obvious suppression of the phenotype was observed for these phosphatase genes in *exoc-8* mutant animals (Figure 2B). These results suggest that the PP2A regulatory subunit *sur-6*/B55 may be involved in regulating *exoc-8* function.

To understand the contribution of the *exoc-8* mutation during early developmental stages in more detail, *exoc-8* animals or animals subjected to RNAi with *sur-6* or *pkc-3* separately, or at the same time were analyzed by using spatio-temporal (4D) microscopy. For this the timing of the embryonic development was measured from the 4-cell to the 28-cell stage, and the percentage of dead or arrested embryos before the pretzel stage was quantified (Figure 2, C and D; Figure S2). Both for wild-type N2 and *exoc-8* mutant animals, it took on average of 70 min to develop from the 4-cell stage to the 28-cell stage (Figure 2C; Figure S2). RNAi with *sur-6* and *pkc-3* caused an additional 173 and 556 min delay, respectively. However, simultaneous down-regulation of these genes reverted the developmental timing to a level that was similar to *exoc-8* animals. It appears that although *sur-6* down-regulation has a weaker effect on developmental timing than *pkc-3* down-regulation, prolonged down-regulation of *sur-6* is significantly more harmful for the overall development of animals (Figure 2D). Taken together, these results support a role for the exocyst complex in the regulation of cell polarity establishment and embryo development in *C. elegans*. Furthermore, the results suggest that a functional link may exist between *sur-6*, *pkc-3*, and *exoc-8* during the early developmental stages.

### The *exoc-8* mutation affects the molecular machinery of the late secretory pathway in *C. elegans*

*C. elegans* with hypomorphic mutations in *exoc-7(ok2006)* and *exoc-8(ok2523)* have defective neuronal functions, and in association with the small GTPase RAB-10 are involved in the recycling of plasma membrane-localized proteins through endocytosis (Jiu *et al.* 2012). In *C. elegans*, the polarized epithelial intestine offers an established model for studying intracellular membrane trafficking and vesicle exocytosis (McGhee 2007). To assess the effect of the RNAi screen hits on vesicular transport, the distribution of several marker proteins for the late secretory pathway were analyzed in N2 wild type and *exoc-8* mutant animals subjected to RNAi.

**Figure 3:**
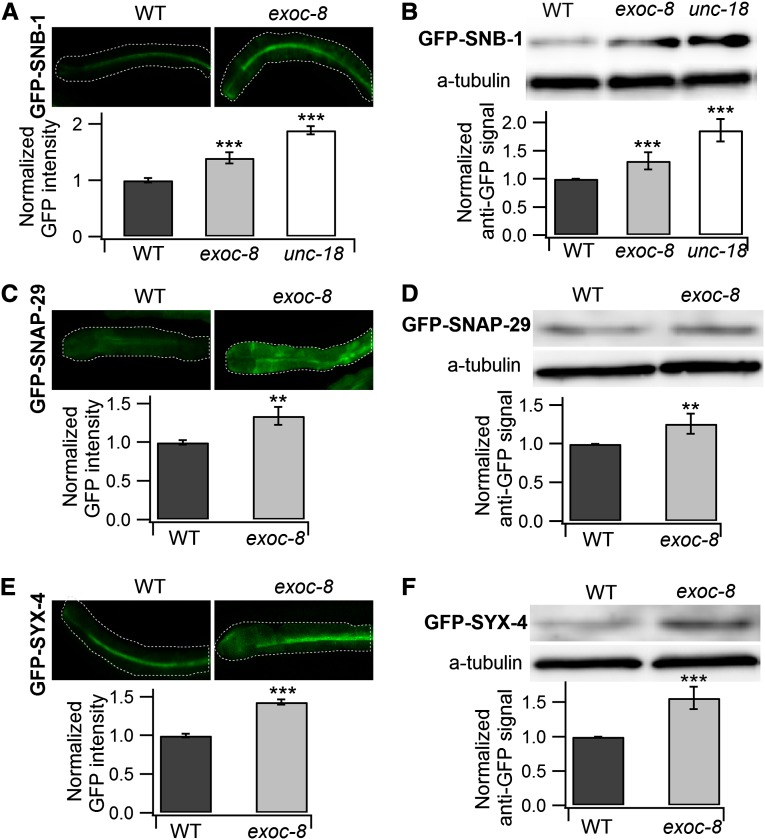
Analysis of membrane traffic components of the late secretory pathway in epithelial intestinal cells in *exoc-8(ok2523)* animals. (A) GFP-SNB-1 fluorescence signal is higher in *exoc-8* and *unc-18* mutants than in wild-type (WT) animals. Lower panel shows the normalized average intensity of GFP fluorescence in transgenic strains carrying pwIs603[P*vha-6*::*GFP*::*SNB-1*] in wild type (n = 36), *exoc-8* (n = 38) and *unc-18* (n = 30) mutant backgrounds. (B) A representative Western blot for GFP-SNB-1 in WT, *exoc-8* and *unc-18* mutants. Lower panel shows the quantification of the GFP-SNB-1 Western blot results from four independent experiments. (C) GFP-SNAP-29 fluorescence signal is higher in *exoc-8* mutants than in WT animals. Lower panel shows the normalized average intensity of GFP fluorescence in transgenic strains carrying pwIs695[P*vha-6*::*GFP*::*SNAP-29*] in WT (n = 32) and *exoc-8* (n = 35) mutant backgrounds. (D) A representative Western blot for GFP-SNAP-29 in WT and *exoc-8* mutants. Lower panel shows the quantification of the GFP-SNAP-29 Western blot results from four independent experiments. (E) GFP-SYX-4 fluorescence signal is higher in *exoc-8* mutants than in WT animals. Lower panel shows the normalized average intensity of GFP fluorescence in transgenic strains carrying pwIs686[P*vha-6*::*GFP*::*SYX-4*] in WT (n = 39) and *exoc-8* (n = 42) mutant mutant backgrounds. (F) A representative Western blot for GFP-SYX-4 in WT and *exoc-8* mutants. Lower panel shows the quantification of the GFP-SYX-4 Western blot results from four independent experiments. In the Western blot α-tubulin was used for normalization. Asterisks denote statistical significance compared with controls, with a *P* value less than 0.01 (**) and 0.001 (***).

The SNARE family proteins are highly conserved core constituents of the protein machinery that facilitate fusion of secretory vesicles within the secretory pathway (Weber *et al.* 1998). Previously, transgenic fusion proteins of SNB-1 (synaptobrevin), SNAP-29 (SNAP-25 family), and SYX-4 (Syntaxin related) fused with GFP at the N-terminus (driven by *vha-6* intestine specific promoter) were localized to the apical surface of the *C. elegans* intestine (Kang *et al.* 2011). When the distribution of ectopically expressed SNB-1, SNAP-29, and SYX-4 (all involved in exocytosis at the plasma membrane) was analyzed in *exoc-8* animals, it was observed that compared with wild-type animals, these SNAREs accumulated within the intestinal epithelium of *exoc-8* mutants (Figure 3, A, C, and E). The enhanced signal was confirmed by Western blot analysis using an anti-GFP antibody (Figure 3, B, D, and F). As a positive control for a defect in exocytosis, *unc-18* mutant animals were used. UNC-18 is the *C. elegans* ortholog of mammalian Munc18 involved in SNARE complex formation during exocytosis. Compared to wild-type animals, increased signal for GFP-SNB-1 was observed in *unc-18(e81)* mutant animals (Figure 3, A and B). The phenotype of accumulated GFP-SNB-1 in *exoc-8* animals resembled greatly that of *unc-18* animals, suggesting that they may cause similar membrane accumulation in cells (Figure 3, A and B; Figure S3). Transgenic worms expressing GFP-SNB-1 and GFP-SNN-1 (synapsin ortholog) driven by the DA motor neuron-specific promoter *unc-129* displayed increased GFP signal in dorsal cord polarized DA neurons (Figure S4). This result is consistent with the observed effects in intestinal cells (Figure 3).

The *C. elegans* IDA-1 protein is closely related to mammalian phogrin that has been employed as a specific dense core vesicle membrane marker (Zahn *et al.* 2001; Cai *et al.* 2004; Zahn *et al.* 2004). To assess the effect of *exoc-8* mutation on an additional marker protein, the level of IDA-1 was investigated. An increased signal for IDA-1-GFP, similarly to SNARE protein markers, was observed in the cell body of the head ALA neuron in *exoc-8* mutants (Figure S5). IDA-1-GFP fluorescence was elevated in a diffused pattern suggesting that IDA-1-GFP is probably distributed in the cytosol (Figure S5). Taken together, these results show that *exoc-8* is functionally important for intracellular vesicular transport.

### *par-1*, *pkc-3*, and *sur-6* are implicated in the regulation of the late secretory pathway in *C. elegans* intestine epithelial cells

**Figure 4:**
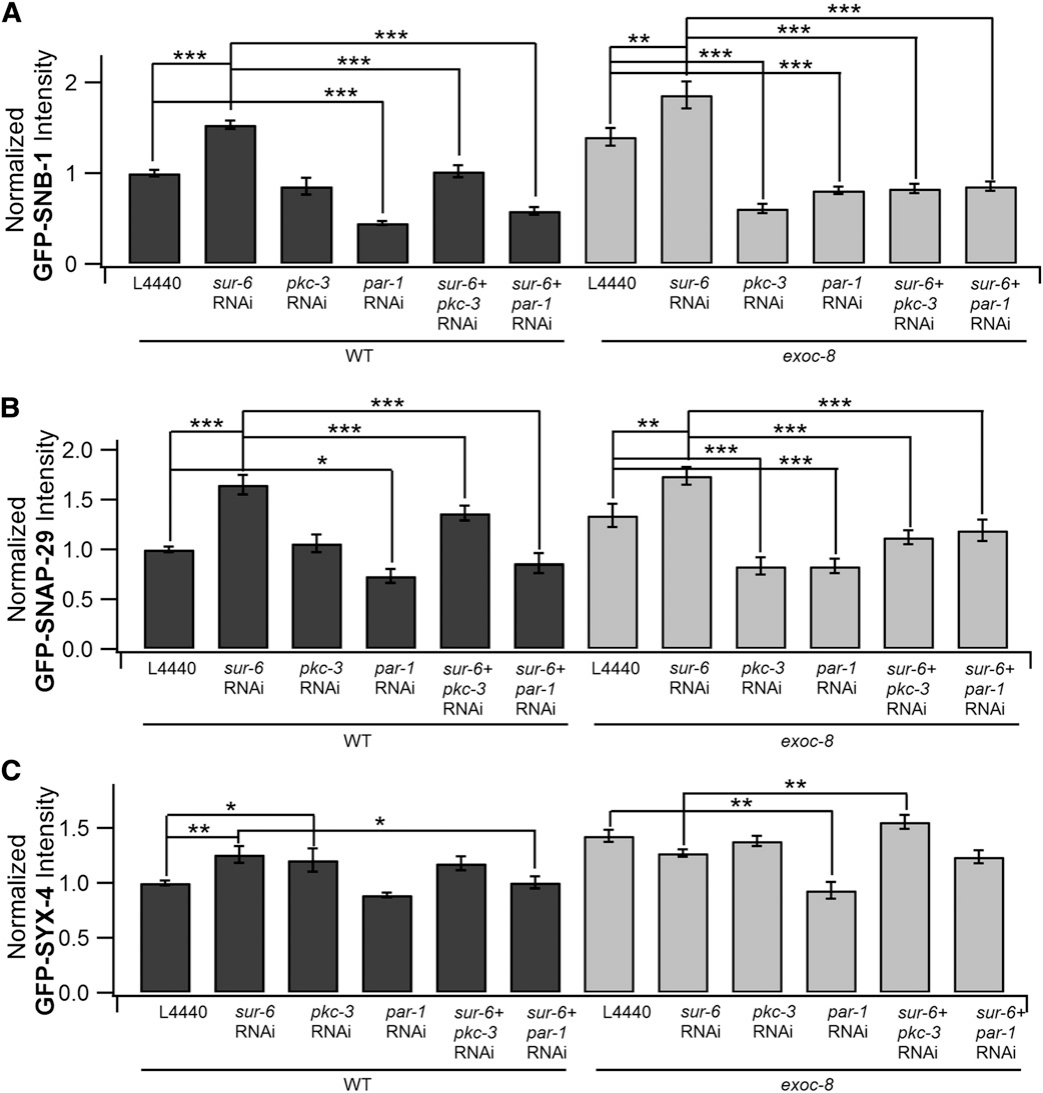
*par-1*, *pkc-3*, and *sur-6* display genetic interactions in the regulation of late secretory pathway marker transport. (A) Normalized average intensity of GFP-SNB-1 fluorescence in L4440, *sur-6(RNAi)*, *pkc-3(RNAi)*, *par-1(RNAi)*, *sur-6(RNAi)*;*pkc-3(RNAi)*, *sur-6(RNAi)*;*par-1(RNAi)*. (B) Normalized average intensity of GFP-SNAP-29 fluorescence in L4440, *sur-6(RNAi)*, *pkc-3(RNAi)*, *par-1(RNAi)*, *sur-6(RNAi);pkc-3(RNAi)*, *sur-6(RNAi);par-1(RNAi)*. (C) Normalized average intensity of GFP-SYX-4 fluorescence in L4440, *sur-6(RNAi)*, *pkc-3(RNAi)*, *par-1(RNAi)*, *sur-6(RNAi);pkc-3(RNAi)*, *sur-6(RNAi);par*-1*(RNAi)*. For each quantification a minimum of 28 animals were used. Asterisks denote statistical significance compared with controls, with a *P* value < 0.05 (*), 0.01 (**), and 0.001 (***).

Having established the *exoc-8* phenotype for several vesicular transport marker proteins, the effect of the identified RNAi screen hits on the same marker proteins was analyzed. Increased levels of GFP-SNB-1 and GFP-SNAP-29 were observed both in wild-type N2 and in *exoc-8* mutants when *sur-6* was knocked-down (Figure 4, A and B, and Figure S6, A and B). In the case of GFP-SYX4, increased GFP signal was observed only in wild-type animals subjected to *sur-6* RNAi (Figure 4C). Knocking down of *pkc-3* did not show any obvious effect on the fluorescence signals for GFP-SNB-1 and GFP-SNAP-29, whereas for GFP-SYX-4 a mild accumulation was observed in the intestine of wild-type animals (Figure 4 and Figure S6). In *exoc-8* mutant animals RNAi of *pkc-3* resulted in reduced fluorescence signal for GFP-SNB-1 and GFP-SNAP-29, while the signal for GFP-SYX-4 was similar to the control cells. With the exception of GFP-SYX-4 in wild-type animals, RNAi of *par-1* caused down-regulation of all SNARE proteins in both wild-type and *exoc-8* backgrounds (Figure 4, Figure S6).

We next tested whether the observed genetic interactions between *par-1*, *pkc-3*, and *sur-6* in *C. elegans* development can be observed at the level of membrane trafficking. For this, simultaneous RNAi of *sur-6* with either *par-1* or *pkc-3* was performed for wild-type N2 and *exoc-8* mutant animals followed by analysis of the distribution of the GFP-tagged marker proteins using confocal microscopy. Both in wild-type and *exoc-8* mutant animals exposed to *sur-6* RNAi, a suppression in GFP-SNB-1 and GFP-SNAP-29 fluorescence signal was detected both for *pkc-3* and *par-1* RNAi (Figure 4, A and B, and Figure S6, A and B). Collectively, these results suggest a functional interplay between *par-1*, *pkc-3*, and *sur-6* in the regulation of SNARE protein transport in intestine epithelial cells in *C. elegans*.

### The exocyst, an aPKC, and PP2A regulate ILP secretion in *Drosophila*

**Figure 5:**
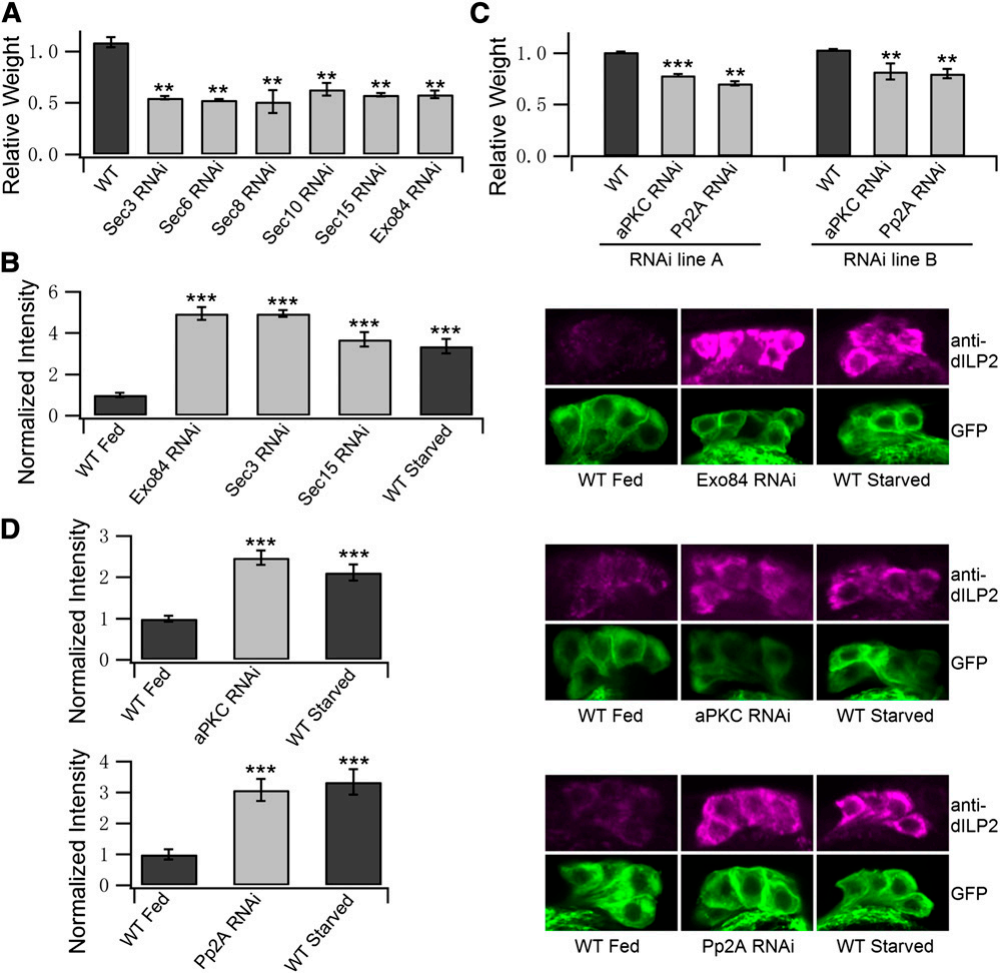
Exocyst complex, aPKC, and Pp2A regulate insulin-like peptide secretion in *Drosophila*. (A) Knockdown of exocyst complex components in IPCs leads to a reduction in body weight. IPC specific knockdown of either Sec3, Sec6, Sec8, Sec10, Sec15, or Exo84 leads to severe reduction in body weight. UAS-RNAi lines were expressed in IPCs using a dILP2-Gal4 driver. dILP2-Gal4 > UAS-GFP was used as a control (WT). (B) Knockdown of exocyst components leads to impaired dILP2 secretion. A dILP2-Gal4 driver was used for IPC-specific knockdown. dILP2-Gal4 > UAS-GFP grown on standard laboratory fly food was used as a control (WT fed). dILP2-Gal4 > UAS-GFP starved on PBS+1% sugar+1% agarose was used as a positive control (WT starved). IPCs are marked by GFP, shown in green and dILP2 immunofluorescence is shown in magenta. Quantified fluorescence intensities for dILP2 in the IPCs of larvae are shown as columns. (C) Knockdown of aPKC or Pp2A in IPCs leads to a reduction in body weight. Two independent RNAi lines were used to knock down aPKC and Pp2A in IPCs using the dILP2-Gal4 driver. dILP2-Gal4 > UAS-GFP was used as a control (WT fed). (D) Knockdown of aPKC or Pp2A leads to impaired dILP2 secretion. Quantified fluorescence intensities for dILP2 in the IPCs of larvae are shown as columns. Knockdown of aPKC and Pp2A leads to increased accumulation of dILP2 in the IPCs. Starvation is used as a positive control. Asterisks denote statistical significance as compared to controls, with a *P* value < 0.01 (**) and 0.001 (***).

To test whether the observed participation in the regulation of protein trafficking by aPKC and PP2A is also observed in other organisms, the secretion of dILPs was analyzed. Exocyst function is required for insulin exocytosis in mammals (Tsuboi *et al.* 2005), but to our knowledge there are no reports of exocyst function for this process in *Drosophila*. *Drosophila* has at least eight dILPs, three of which (dILP2, -3, and -5) are secreted by the median neurosecretory cells, also known as IPCs (Rulifson *et al.* 2002). Because dILPs are critical regulators of growth (Ikeya *et al.* 2002; Rulifson *et al.* 2002), the function of IPC can be indirectly assessed by analyzing total body weight after IPC-specific gene manipulation. Knockdown of core components of the exocyst (Sec3, Sec6, Sec8, Sec10, Exo84, or Sec15) specifically in IPCs leads to >40% reduction in body weight (Figure 5A), implying that exocyst has a critical role in IPC function.

Accumulation of dILPs into the IPC cell bodies is a well-established consequence of inhibited dILP secretion, *e.g.*, upon nutrient starvation (Geminard *et al.* 2009). This can be detected using immunofluorescence confocal microscopy. Knock-down of either Exo84, Sec3, or Sec15 led to strong accumulation of dILP2 in cell bodies of IPCs (Figure 5B), suggesting that exocyst function is necessary for dILP2 secretion in the IPCs of *Drosophila* larvae. Starvation of the larvae was used as a positive control.

To explore the possible functional conservation of the exocyst regulators identified in *C. elegans*, their role for *Drosophila* dILP secretion was analyzed. *Drosophila* aPKC (CG42783) and PP2A-29B (CG17291), orthologs of the *C. elegans pkc-3* and *paa-1* genes identified in our screen, displayed an essential function in *Drosophila* IPCs. IPC-specific knockdown of aPKC and PP2A-29B with two independent RNAi lines led to a significant reduction of total body weight (Figure 5C). To directly explore the effect of these genes on dILP secretion, we analyzed the dILP2 accumulation by immunofluorescence. The accumulation observed upon knockdown of either aPKC or Pp2A-29B was comparable to the accumulation observed in starved larvae, which were our positive controls (Figure 5D). In conclusion, our data shows that dILP secretion is exocyst-dependent, and both aPKC and PP2A have an essential role in this process.

## Discussion

There is a large body of evidence linking small GTPases to the functional regulation of the exocyst complex (Mizuno-Yamasaki *et al.* 2012). At the same time, very few data exist on the contribution of phosphorylation-dephosphorylation cycles on exocyst regulation. In the present study, we set out to identify kinases and phosphatases that are functionally linked with the exocyst. For this a targeted RNAi screen was carried out in *C. elegans* animals mildly defective in the exocyst subunits Exo70 and Exo84. The screen identified seven kinases and seven phosphatase genes, down-regulation of which caused a variable degree of loss of viability when combined with exocyst mutations. For *kin-20*, the seventh kinase hit, a surprising partial rescue of the severely sick phenotype was observed in *exoc-7* animals, and a full rescue was seen in *exoc-8* mutants and in *exoc-7*; *exoc-8* double mutants. *kin-20* is the *C. elegans* homolog of the *Drosophila* circadian clock gene *doubletime* that is required to maintain late-larval identity and prevents premature expression of adult cell fates (Kloss *et al.* 1998). Despite the strong genetic interaction with *exoc* mutants the functional relationship between these genes is presently unclear.

Additional hits in the kinase screen included *let-23*, *par-1*, *pkc-3*, *tpa-1*, and *B0252.1* (Table 1). *tpa-1* encodes two protein kinase C isoforms, TPA-1A and TPA-1B. The analysis of *tpa-1* mutations indicates that at least one TPA-1 isoform plays a role in nicotine-induced adaptation and that both isoforms contribute to G protein-mediated signaling that modulates feeding and growth (van der Linden *et al.* 2003). The putative homolog Pkc1 in *S. cerevisiae* is a serine/threonine kinase essential for cell wall remodeling during growth, and it is localized to sites of polarized growth. Such a distribution is similar to that reported for the exocyst complex in yeast (TerBush *et al.* 1996). *let-23* encodes an EGF-receptor-family *trans*-membrane tyrosine kinase that has been shown to affect viability, and, *e.g.*, inductive signaling during development of the vulval precursor cells (Aroian *et al.* 1994). It can be speculated that in *exoc-7*; *exoc-8* double-mutant animals a mild transport defect for correct LET-23 plasma membrane targeting exists. Upon down-regulation of LET-23 by RNAi insufficient amounts of LET-23 could reach the target membrane and a growth defect would be observed. Interestingly, a RAB-family small GTPase, RAB-7, was recently implicated in recycling of LET-23 from the cell surface to the endosomal compartment (Skorobogata and Rocheleau 2012). Similarly, we have shown that in *exoc-7;exoc-8* double-mutant animals defective recycling of endosomal marker proteins is observed (Jiu *et al.* 2012). This finding suggests that the genetic interactions observed for *let-23* may be caused by its defective transport in exocyst mutants. *B0252.1* is a putative homolog of Cla4, a Cdc42-activated signal transducing kinase of the PAK (p21-activated kinase) family in *S. cerevisiae* that is functionally important for cell polarity generation. However, no previous data exist on a close functional link with the exocyst. The synthetic lethality observed here between B0252.1(gk1114) and exoc-7;exoc-8 mutations lends support for a functional interaction.

The genetic interactions observed in our study are in agreement with studies in yeast, where the PAR-1 homologs Kin1p and Kin2p have been shown to interact with the SNARE protein Sec9p and the LGL homolog Sro7p (Elbert *et al.* 2005). Phosphorylation by Kin1p/Kin2p caused the release of Sec9p from the membrane (Elbert *et al.* 2005). Interestingly, it was shown that overexpression of the kinase domain of Kin2p was able to rescue the temperature sensitive growth defect of cells bearing mutations in the exocyst components Sec15p and Sec10p, as well as Sec2p, which is the guanine exchange factor for the Sec15p-interacting RAB GTPase Sec4p. Another study showed that Sec4p phosphorylation appears to negatively regulate the interaction of Sec4p with its effector Sec15p, and that Cdc55p, the homolog of *C. elegans* SUR-6, is the responsible phosphatase for Sec4p implicated in this process (Heger *et al.* 2011).

The kinase screen hits *par-1* and *pkc-3* belong to the major cell polarization regulator PAR protein family. When the combined effects for the simultaneous RNAi of identified kinase and phosphatase hits were analyzed, a genetic interaction between the regulatory subunit of PP2A and *par-1* and *pkc-3* was observed. In *C. elegans*, *paa-1* and *let-92* are the only genes encoding the structural A subunit and the catalytic C subunit, respectively. The B subunit variants are encoded by seven genes belonging to three families (B55, B56, and B72) in *C. elegans*, and *sur-6*, identified in our screen, is the only representative of the *C. elegans* B55 family. *sur-6*/B55 is involved in the regulation of several cellular processes in different model systems, such as oncogenesis by the AKT pathway (Kuo *et al.* 2008), vulva development in *C. elegans*, where it inhibits PAR-1 (Hurd and Kemphues 2003), embryonic development (Kao *et al.* 2004), centriole duplication (Song *et al.* 2011), and axon guidance (Ogura *et al.* 2010). The specificity of the genetic interaction between *sur-6*, *par-1*, and *pkc-3* in *C. elegans* is supported by the fact that down-regulation of the other PP2A regulatory subunit family members did not result in reduced viability of *exoc* mutant animals (Figure 2). Importantly, a similar apparently counteractive interdependence was observed when trafficking of exocytic membrane anchored SNARE proteins were investigated in intestine epithelial cells (Figure 4). However, based on these results specific mechanistic explanations for the observed genetic interactions cannot be drawn. Nevertheless, our results suggest that *sur-6* may be directly or indirectly involved in the regulation of protein transport and exocyst function.

On the basis of the genes identified in *C. elegans*, we performed a targeted RNAi analysis of genes affecting *Drosophila* dILP secretion, which we show to be an exocyst-dependent process. The results indicate an essential function for the exocyst in dILP secretion and consequent systemic regulation of body weight. Furthermore, they show that interference of aPKC and PP2A function leads to strongly impaired dILP2 secretion. The IPCs are neuroendocrine cells with cell bodies localized within the *pars intercerebralis* and long processes terminating in the heart and the ring gland. Immunolabeling has shown that dILP2 is localized in these processes (Rulifson *et al.* 2002). Since the exocyst complex is known to tether secretory vesicles to specific plasma membrane sites, it might be involved in the localization of dILP for exocytosis. However, our immunostainings did not have sufficient sensitivity and resolution to address this. The data presented in this study show that both aPKC and PP2A have an essential role in dILP secretion in the IPCs, and together with the *C. elegans* results suggest that the involvement of these regulators in protein trafficking may be conserved across species. Clearly, further studies are needed to address how related kinases and phosphatases contribute to the protein secretion process in different species.

Our results identify kinase and phosphatase genes that are functionally implicated in the molecular processes in which the exocyst complex is known to operate. In mammalian cells binding of the RalA GTPase to the exocyst subunit Sec5 was shown to be dependent on the phosphorylation of Sec5 (Chen *et al.* 2011a). Sec5 phosphorylation is carried out by PKC, and this phosphorylation appears to cause detachment of Sec5 from RalA, which is bound to the transport vesicle. This in turn enables the transport vesicle to proceed for fusion with the plasma membrane (Chen *et al.* 2011a,b). The phosphatase responsible for Sec5 dephosphorylation and subsequent reassociation of Sec5 with RalA for another round of vesicle fusion has remained unindentified. It is tempting to speculate that a PP2A containing a B55 family member regulatory subunit may be responsible for the dephosphorylation of Sec5p also in mammalian cells (Chen *et al.* 2011a). A strong functional link in hypodermal cell organization regulation has been shown for *C. elegans* Ral GTPase and the exocyst (Frische *et al.* 2007). It is therefore possible that similarly to mammalian cells a regulatory process for Ral-exocyst interaction operates through a phosphorylation-dephosphorylation cycle in *C. elegans*. However, it should be noted that the reported phosphorylation site in Sec5 (Ser89) appears not to be conserved in the Ral-binding domains of *C. elegans* and *Drosophila* Sec5. It is therefore possible that a different phosphorylation site or even different kinases and phosphatases are required in these organisms for the regulation of Sec5. Taken together, the present study identifies a set of kinases and phosphatases that offer interesting leads for future studies into the regulation of the exocyst complex.

## Supplementary Material

Supporting Information
