# Supplementary material for: par-1, Atypical pkc, and PP2A/B55 sur-6 Are Implicated in the Regulation of Exocyst-Mediated Membrane Trafficking in Caenorhabditis elegans
Source: G3 (Bethesda). 2013 Nov 5;4(1):173–83. doi: 10.1534/g3.113.006718 (PMC3887533; doi:10.1534/g3.113.006718)
Supplement: Supporting Information [file supp_4_1_173__index.html]

par-1, Atypical pkc, and PP2A/B55 sur-6 Are Implicated in the Regulation of Exocyst-Mediated Membrane Trafficking in Caenorhabditis elegans — Supporting Information 

# *par-1*, Atypical *pkc*, and PP2A/B55 *sur-6* Are Implicated in the Regulation of Exocyst-Mediated Membrane Trafficking in *Caenorhabditis elegans*

## Supporting Information for Jiu *et al.*, 2014

**Files in this Data Supplement:**

- Supporting Information - Figures S1-S6 and Table S1 (PDF, 1 MB)
- Figure S1 - qRT-PCR quantification of the RNA silencing efficiency for a set of the candidate genes in *rrf-3* worms. (PDF, 397 KB)
- Figure S2 - Representative images of embryo development in different time points in wild type N2 and *exoc-8* mutants with control, *sur-6(RNAi), pkc-3(RNAi)*, and *sur-6(RNAi);pkc-3(RNAi)*, respectively. (PDF, 608 KB)
- Figure S3 - GFP-SNB-1 expression pattern in epithelial intestine in wild type (WT) and *unc-18*(*e81*) mutant animals. (PDF, 348 KB)
- Figure S4 - SNB-1 and SNN-1 are accumulated in DA motor neurons in *exoc-8* mutants. (PDF, 396 KB)
- Figure S5 - IDA-1 expression in ALA neuron in both wild type (WT) and *exoc-8* mutants. (PDF, 428 KB)
- Figure S6 - Representative figures of the genetic interations observed between *par-1, pkc-3 sur-6* RNAi-for the localization of late secretory pathway markers. (PDF, 443 KB)
- Table S1 - Oligo sequences for qRT-PCR. (PDF, 312 KB)
