# Supplementary material for: par-1, Atypical pkc, and PP2A/B55 sur-6 Are Implicated in the Regulation of Exocyst-Mediated Membrane Trafficking in Caenorhabditis elegans
Source: G3 (Bethesda). 2013 Nov 5;4(1):173–83. doi: 10.1534/g3.113.006718 (PMC3887533; doi:10.1534/g3.113.006718)
Supplement: Supporting Information [file supp_g3.113.006718_006718SI.pdf]

G3 · Genes | Genomes | Genetics

par-1, atypical *pkc* and PP2A/B55 *sur-6* are implicated in the regulation of exocyst-mediated membrane trafficking in *C. elegans*

Yaming Jiu*, Kiran Hasygar*,§, Lois Tang†, Yanbo Liu*, Carina I. Holmberg‡, Thomas R. Bürglin†, Ville Hietakangas*,§ and Jussi Jäntti*,**

* Institute of Biotechnology, Research program in Cell and Molecular Biology, P.O. Box 56, 00014 University of Helsinki, Finland
§ Department of Biosciences, University of Helsinki, Finland
† Department of Biosciences and Nutrition, and Center for Biosciences, Karolinska Institutet, Novum, SE 141 83, Huddinge, Sweden
‡ Research Programs Unit, Translational Cancer Biology, and Institute of Biomedicine, P.O. Box 63, 00014 University of Helsinki, Finland
** VTT Technical Research Centre of Finland, P.O. Box 1000, Espoo, 02044 VTT, Finland

Corresponding author:
Jussi Jäntti, PhD, VTT Technical Research Centre of Finland, PO Box 1000 (Street address: Tietotie 2), FIN-02044 VTT, Finland
Tel: +358 40 199 3556/+358 50 522 7846
email: jussi.jantti@vtt.fi

DOI: 10.1534/g3.113.006718

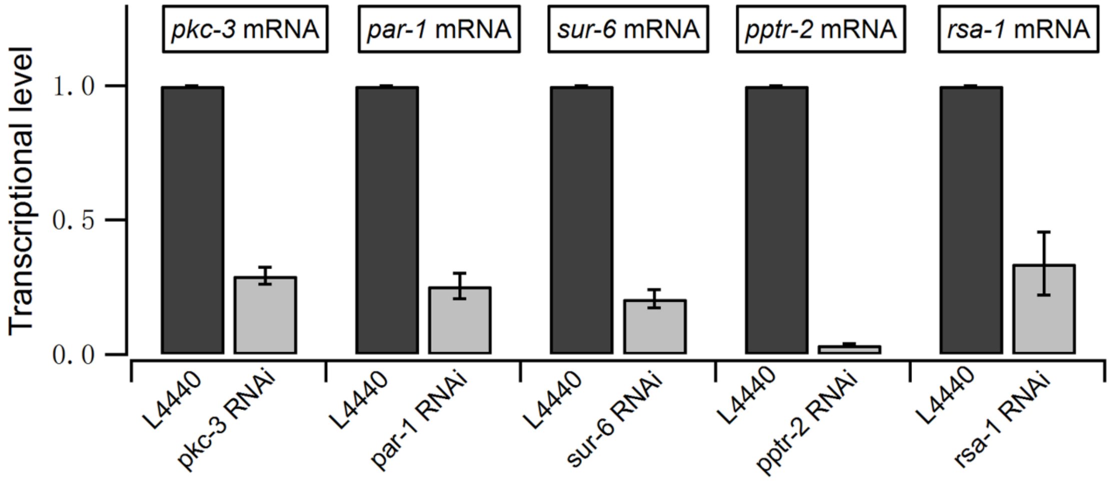

Figure S1 qRT-PCR quantification of the RNA silencing efficiency for a set of the candidate genes in *rrf-3* worms. qRT-PCR quantification of the RNA silencing efficiency for *pkc-3, par-1, sur-6, pptr-2 and rsa-1* in *rrf-3* worms. The mRNA levels of controls were set as arbitrary unit 1. α- tubulin mRNA was used for signal normalization.

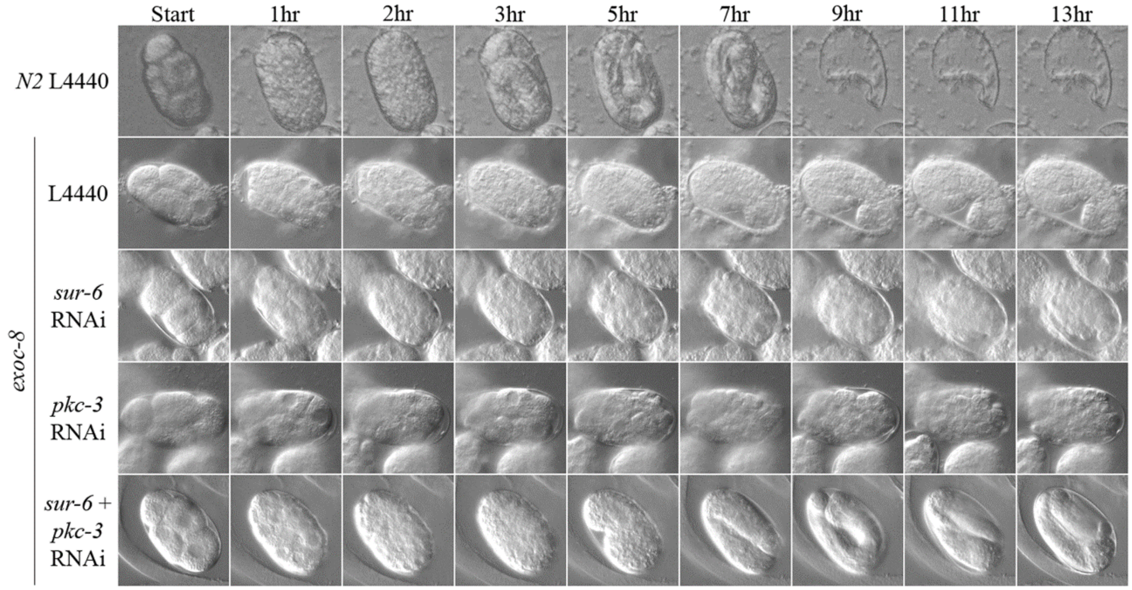

Figure S2 Representative images of embryo development in different time points in wild type N2 and *exoc-8* mutants with control, *sur-6(RNAi)*, *pkc-3(RNAi)*, and *sur-6(RNAi);pkc-3(RNAi)*, respectively.

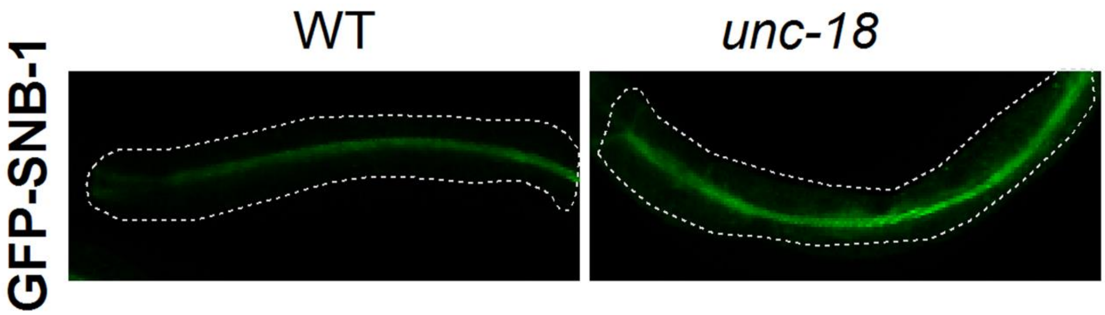

Figure S3 GFP-SNB-1 expression pattern in epithelial intestine in wild type (WT) and *unc-18*(*e81*) mutant animals.

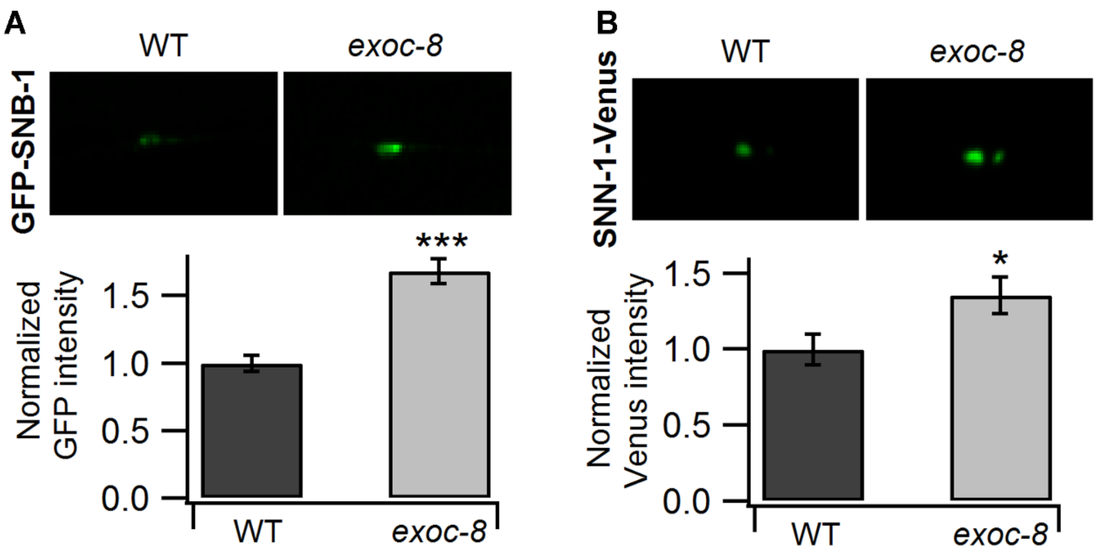

Figure S4 SNB-1 and SNN-1 are accumulated in DA motor neurons in *exoc-8* mutants. (A) GFP-SNB-1 fluorescence signal is increased in DA motor neurons of *exoc-8* mutant animals. Lower panel shows the normalized average intensity of GFP fluorescence in transgenic strains nuIs152[P*unc-129::GFP::SNB-1*] in wild-type (n=39) and *exoc-8* mutant(n=43) backgrounds. (B) SNN-1-GFP fluorescence is increased in *exoc-8* mutants. Lower panel shows the normalized average intensity of GFP fluorescence in transgenic strains nuIs163[P*unc-129::SNN-1::Venus*] in wild-type (n=34) and *exoc-8* mutant (n=38) background. Asterisks denote statistical significance as compared to controls, with a P value less than 0.05 (*), and 0.001 (***).

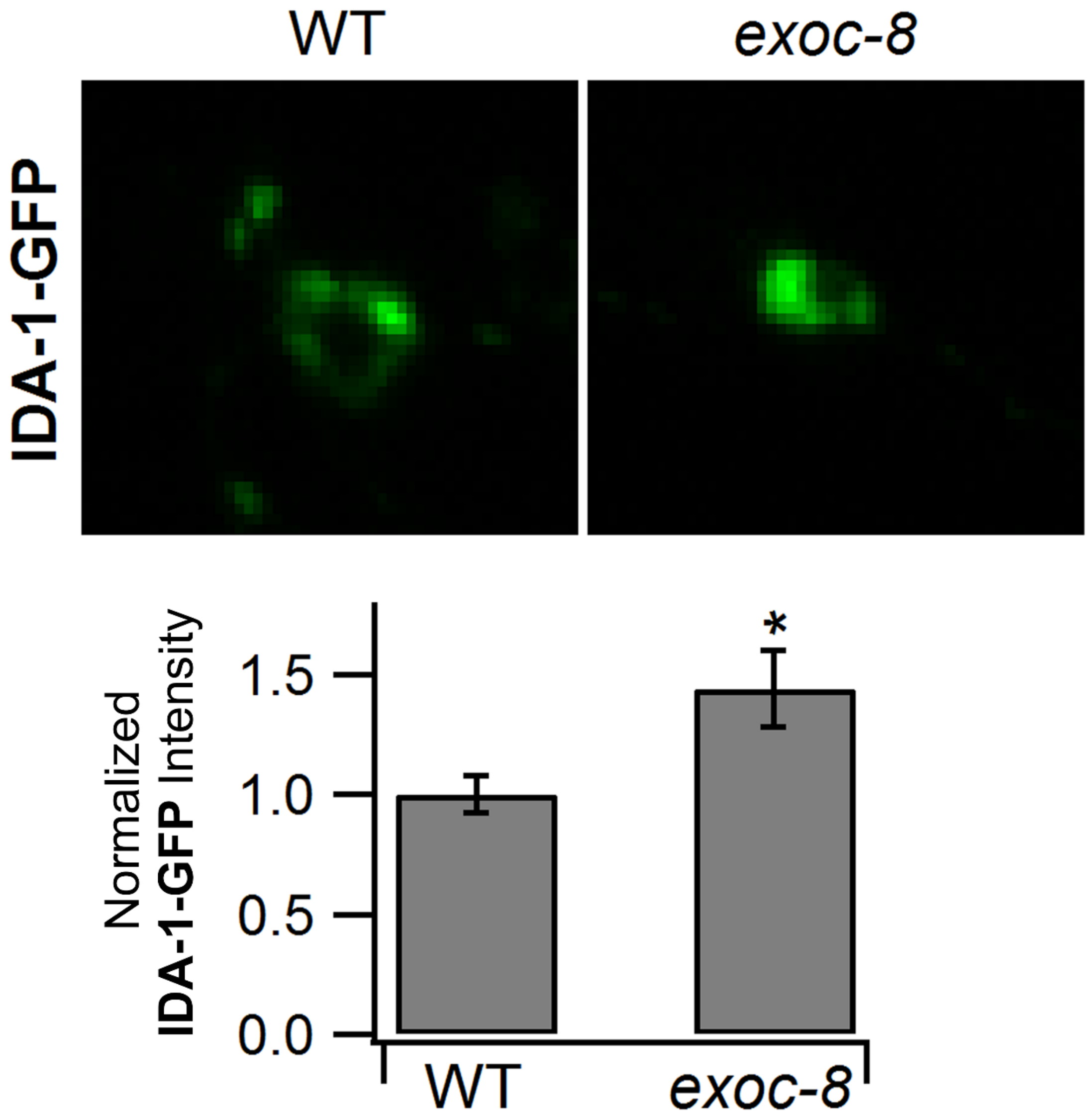

Figure S5 IDA-1 expression in ALA neuron in both wild type (WT) and *exoc-8* mutants. IDA-1-GFP fluorescence is increased in *exoc-8* mutants. Lower panel shows the normalized average intensity of GFP fluorescence in WT (n=45) and *exoc-8* mutant (n=41) backgrounds. Asterisks denote statistical significance as compared to controls, with a P value less than 0.05 (*).

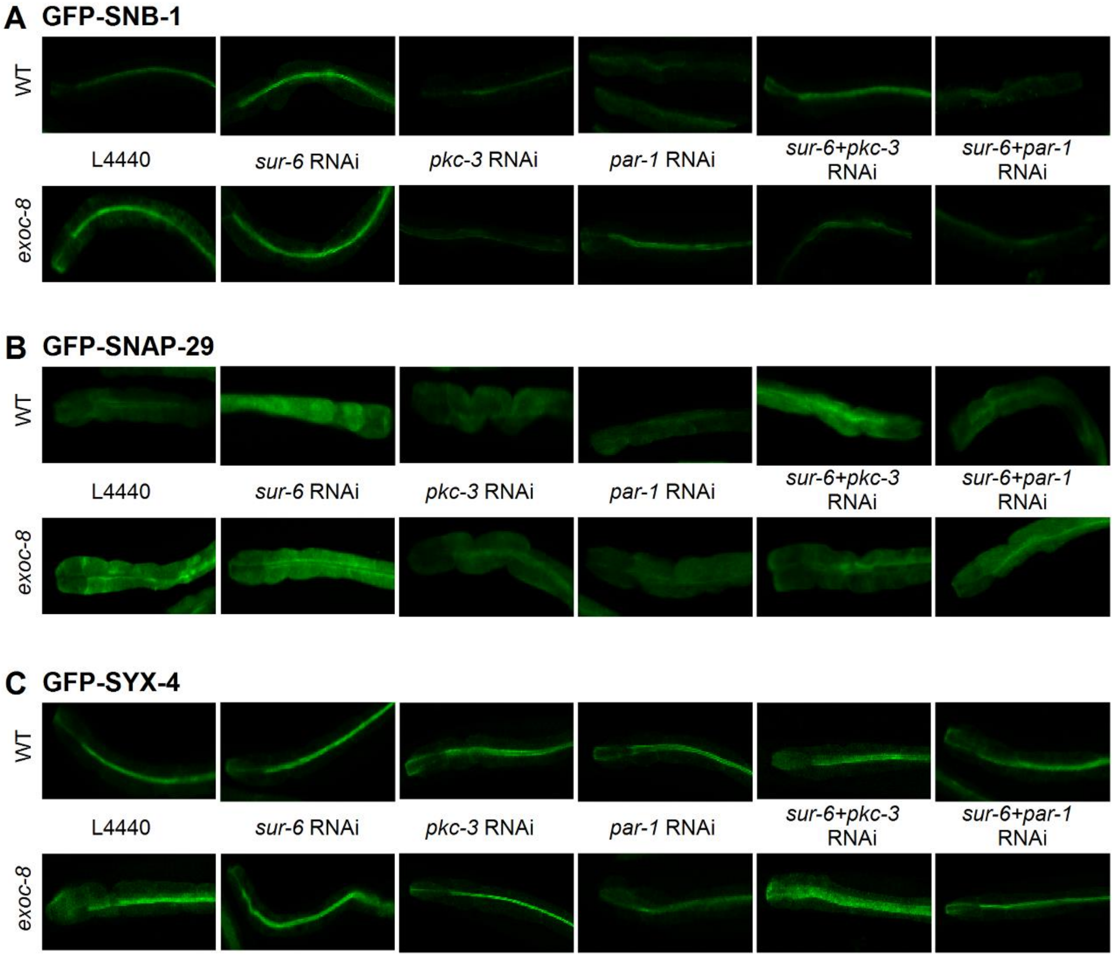

Figure S6 Representative figures of the genetic interations observed between *par-1, pkc-3 sur-6* RNAi-for the localization of late secretory pathway markers. (A) Localization of GFP-SNB-1 fluorescence in WT and *exoc-8* mutant animals treated with L4440, *sur-6(RNAi)*, *pkc-3(RNAi)*, *par-1(RNAi)*, *sur-6(RNAi);pkc-3(RNAi)*, *sur-6(RNAi);par-1(RNAi)*. (B) Localization of GFP-SNAP-29 fluorescence in WT and *exoc-8* mutant animals treated with L4440, *sur-6(RNAi)*, *pkc-3(RNAi)*, *par-1(RNAi)*, *sur-6(RNAi);pkc-3(RNAi)*, *sur-6(RNAi);par-1(RNAi)*. (C) Localization of GFP-SYX-4 fluorescence in WT and *exoc-8* mutant animals treated with L4440, *sur-6(RNAi)*, *pkc-3(RNAi)*, *par-1(RNAi)*, *sur-6(RNAi)*;*pkc-3(RNAi)*, *sur-6(RNAi);par-1(RNAi)*.

Table S1 Oligo sequences for qRT-PCR

oligo name	oligo sequence
*pkc-3*_forward	5’ CCATCGGCATGTGCGAACGCA 3’
*pkc-3*_reverse	5’ TCCTCCACCCGACCGCGTTG 3’
*par-1*_forward	5’ GGAGCAACTGGTCCATCGGCCA 3’
*par-1*_reverse	5’ GCACGTCCTGATACTGGCTGGGT 3’
*sur-6*_forward	5’ CCTATCTGCCGACGATTTGCGAGT 3’
*sur-6*_reverse	5’ TGCGTTGGATGGAATTCGGCG 3’
*pptr-2*_forward	5’ CGTCGACCACGTTTCCGGAGC 3’
*pptr-2*_reverse	5’ TGCAGATGTGGCCAGGCAGC 3’
*rsa-1*_forward	5’ TGTTGGCGCCAGCGGACTTG 3’
*rsa-2*_reverse	5’ TGGCGGAGGCAGTGGTATGACG 3’
