# Supplementary material for: par-1, Atypical pkc, and PP2A/B55 sur-6 Are Implicated in the Regulation of Exocyst-Mediated Membrane Trafficking in Caenorhabditis elegans
Source: G3 (Bethesda). 2013 Nov 5;4(1):173–83. doi: 10.1534/g3.113.006718 (PMC3887533; doi:10.1534/g3.113.006718)
Supplement: Supporting Information [file supp_g3.113.006718_FigureS1.pdf]

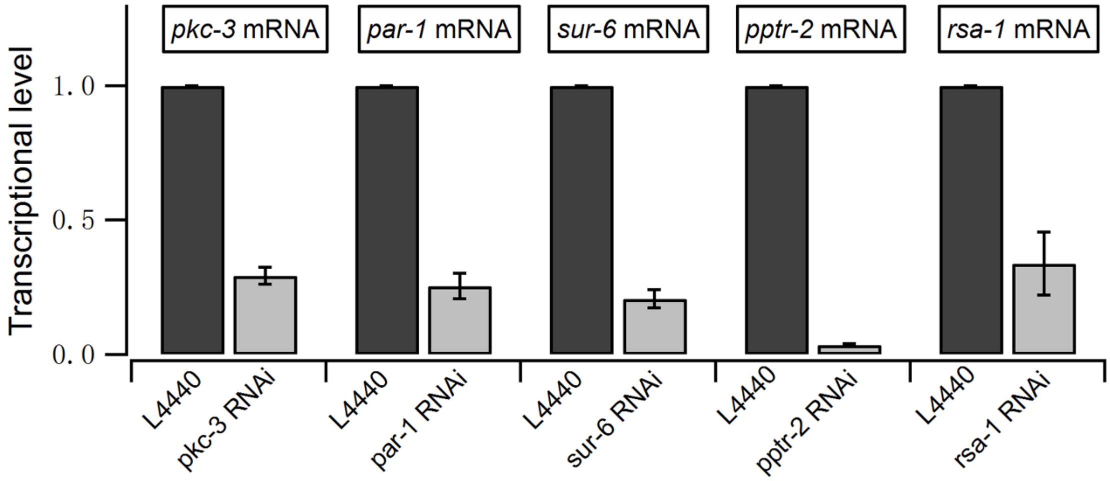


**Figure S1** qRT-PCR quantification of the RNA silencing efficiency for a set of the candidate genes in *rrf-3* worms. qRT-PCR quantification of the RNA silencing efficiency for *pkc-3, par-1, sur-6, pptr-2 and rsa-1* in *rrf-3* worms. The mRNA levels of controls were set as arbitrary unit 1. α- tubulin mRNA was used for signal normalization.
