# Supplementary material for: par-1, Atypical pkc, and PP2A/B55 sur-6 Are Implicated in the Regulation of Exocyst-Mediated Membrane Trafficking in Caenorhabditis elegans
Source: G3 (Bethesda). 2013 Nov 5;4(1):173–83. doi: 10.1534/g3.113.006718 (PMC3887533; doi:10.1534/g3.113.006718)
Supplement: Supporting Information [file supp_g3.113.006718_FigureS2.pdf]

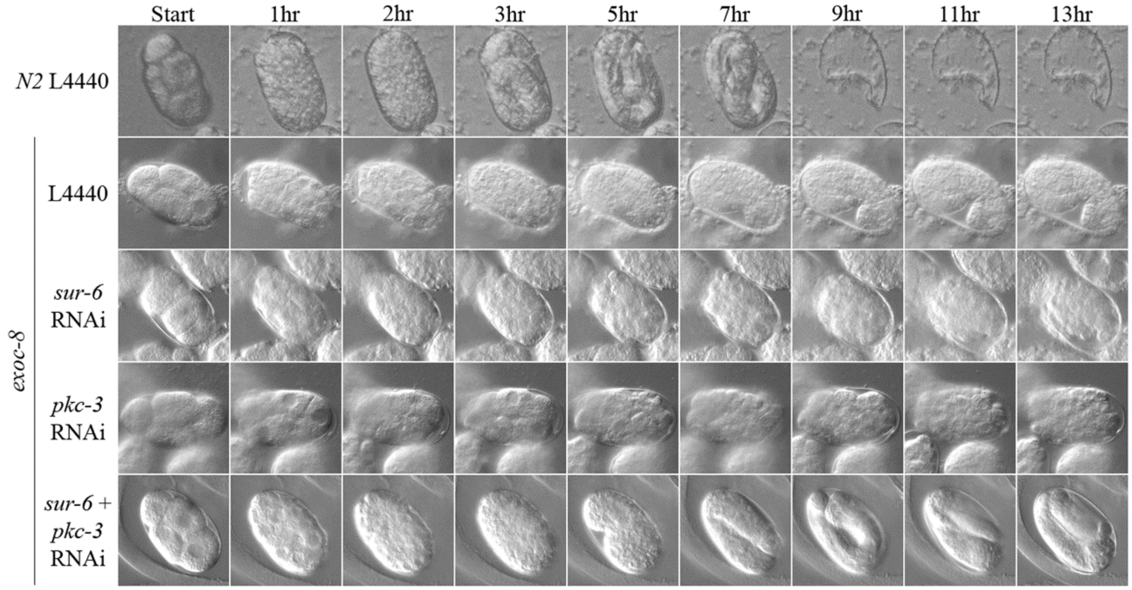

Figure S2 Representative images of embryo development in different time points in wild type N2 and *exoc-8* mutants with control, *sur-6(RNAi)*, *pkc-3(RNAi)*, and *sur-6(RNAi);pkc-3(RNAi)*, respectively.
