# Supplementary material for: par-1, Atypical pkc, and PP2A/B55 sur-6 Are Implicated in the Regulation of Exocyst-Mediated Membrane Trafficking in Caenorhabditis elegans
Source: G3 (Bethesda). 2013 Nov 5;4(1):173–83. doi: 10.1534/g3.113.006718 (PMC3887533; doi:10.1534/g3.113.006718)
Supplement: Supporting Information [file supp_g3.113.006718_FigureS4.pdf]

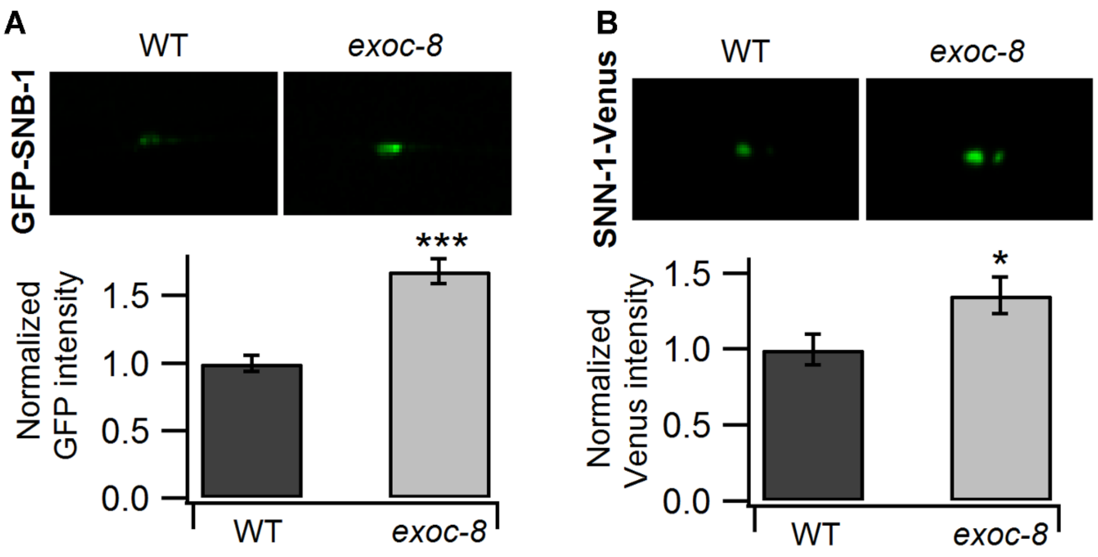

Figure S4 SNB-1 and SNN-1 are accumulated in DA motor neurons in *exoc-8* mutants. (A) GFP-SNB-1 fluorescence signal is increased in DA motor neurons of *exoc-8* mutant animals. Lower panel shows the normalized average intensity of GFP fluorescence in transgenic strains nuIs152[P*unc-129::GFP::SNB-1*] in wild-type (n=39) and *exoc-8* mutant(n=43) backgrounds. (B) SNN-1-GFP fluorescence is increased in *exoc-8* mutants. Lower panel shows the normalized average intensity of GFP fluorescence in transgenic strains nuIs163[P*unc-129::SNN-1::Venus*] in wild-type (n=34) and *exoc-8* mutant (n=38) background. Asterisks denote statistical significance as compared to controls, with a P value less than 0.05 (*), and 0.001 (***).
