# Supplementary material for: par-1, Atypical pkc, and PP2A/B55 sur-6 Are Implicated in the Regulation of Exocyst-Mediated Membrane Trafficking in Caenorhabditis elegans
Source: G3 (Bethesda). 2013 Nov 5;4(1):173–83. doi: 10.1534/g3.113.006718 (PMC3887533; doi:10.1534/g3.113.006718)
Supplement: Supporting Information [file supp_g3.113.006718_FigureS5.pdf]

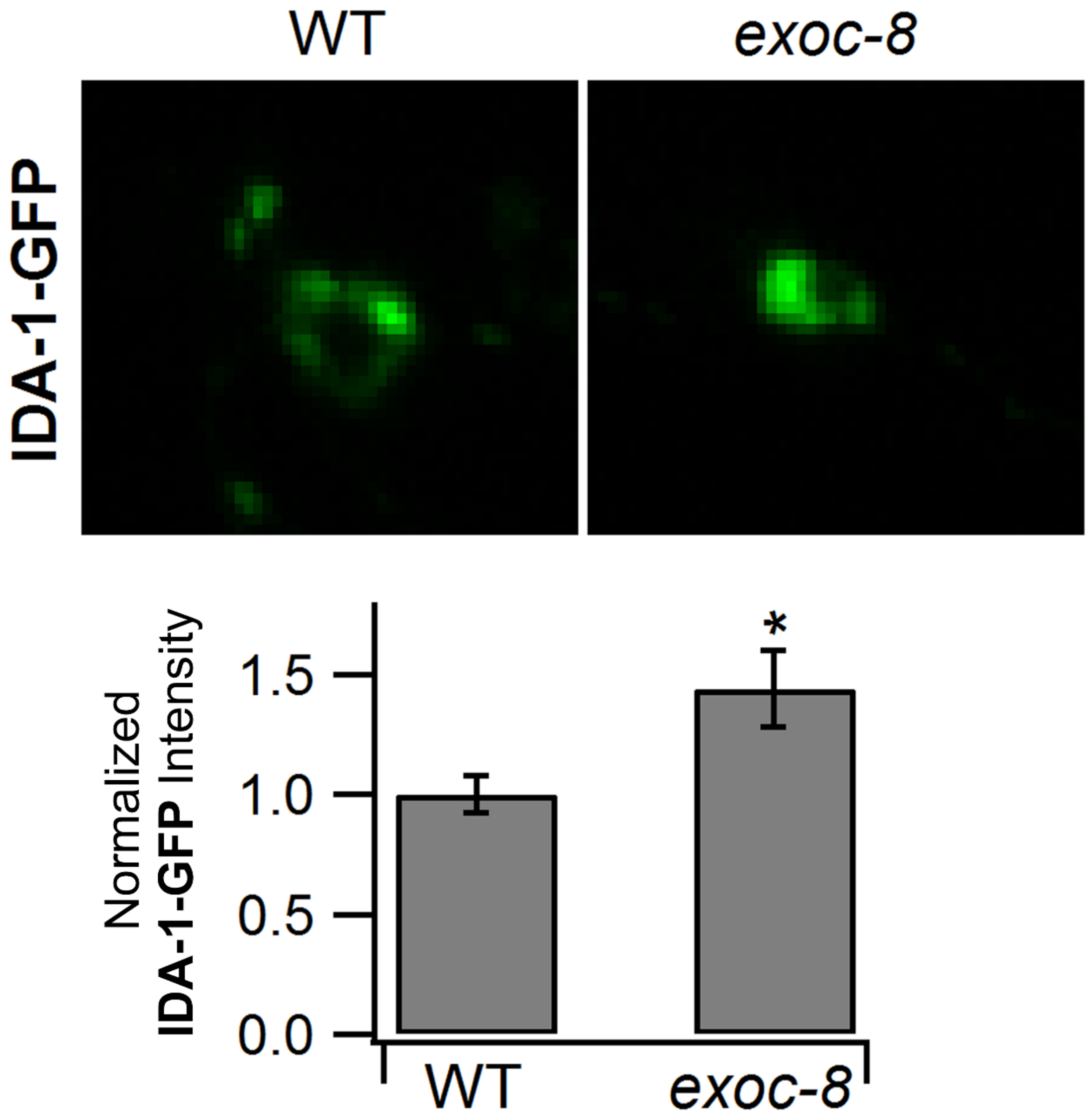

Figure S5 IDA-1 expression in ALA neuron in both wild type (WT) and *exoc-8* mutants. IDA-1-GFP fluorescence is increased in *exoc-8* mutants. Lower panel shows the normalized average intensity of GFP fluorescence in WT (n=45) and *exoc-8* mutant (n=41) backgrounds. Asterisks denote statistical significance as compared to controls, with a P value less than 0.05 (*).
