# Supplementary material for: par-1, Atypical pkc, and PP2A/B55 sur-6 Are Implicated in the Regulation of Exocyst-Mediated Membrane Trafficking in Caenorhabditis elegans
Source: G3 (Bethesda). 2013 Nov 5;4(1):173–83. doi: 10.1534/g3.113.006718 (PMC3887533; doi:10.1534/g3.113.006718)
Supplement: Supporting Information [file supp_g3.113.006718_FigureS6.pdf]

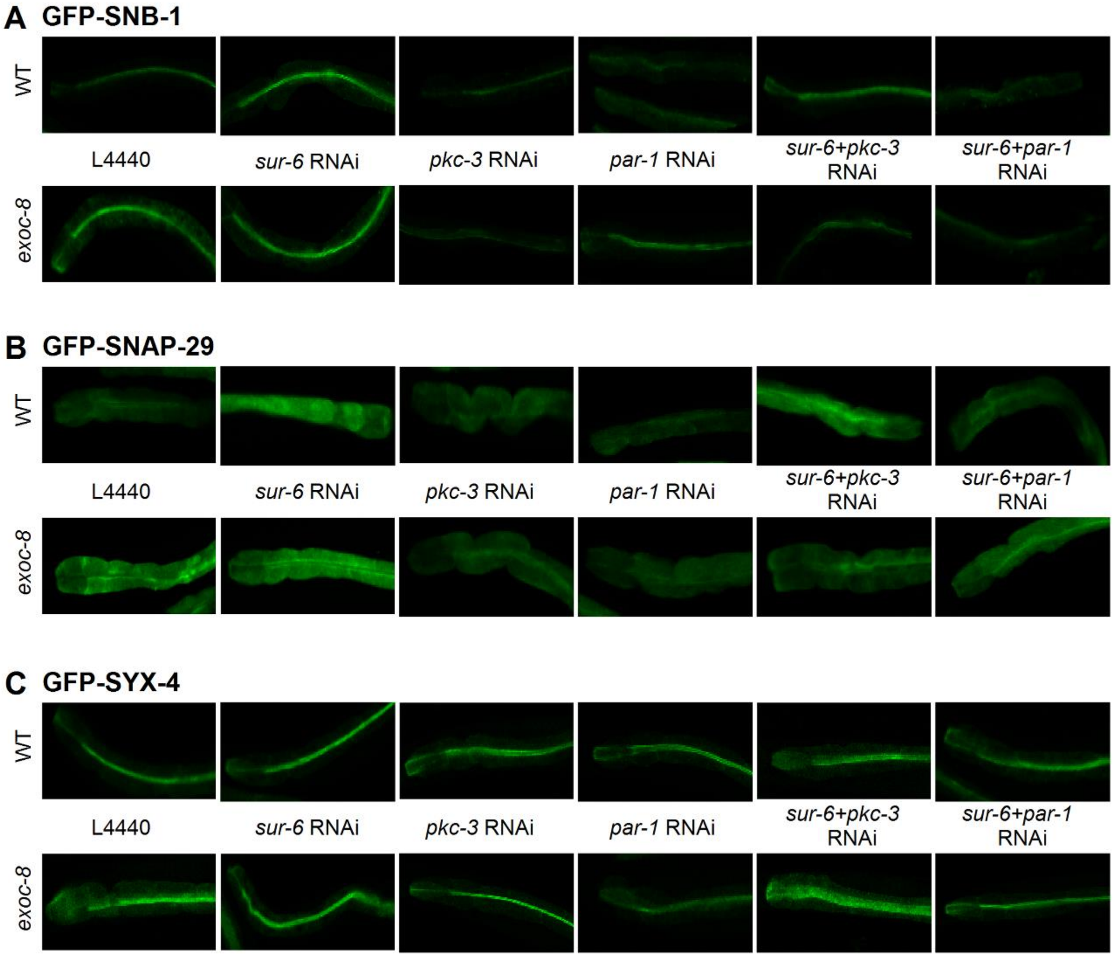

Figure S6 Representative figures of the genetic interations observed between *par-1, pkc-3 sur-6* RNAi-for the localization of late secretory pathway markers. (A) Localization of GFP-SNB-1 fluorescence in WT and *exoc-8* mutant animals treated with L4440, *sur-6(RNAi)*, *pkc-3(RNAi)*, *par-1(RNAi)*, *sur-6(RNAi);pkc-3(RNAi)*, *sur-6(RNAi);par-1(RNAi)*. (B) Localization of GFP-SNAP-29 fluorescence in WT and *exoc-8* mutant animals treated with L4440, *sur-6(RNAi)*, *pkc-3(RNAi)*, *par-1(RNAi)*, *sur-6(RNAi);pkc-3(RNAi)*, *sur-6(RNAi);par-1(RNAi)*. (C) Localization of GFP-SYX-4 fluorescence in WT and *exoc-8* mutant animals treated with L4440, *sur-6(RNAi)*, *pkc-3(RNAi)*, *par-1(RNAi)*, *sur-6(RNAi)*;*pkc-3(RNAi)*, *sur-6(RNAi);par-1(RNAi)*.
