# Supplementary material for: par-1, Atypical pkc, and PP2A/B55 sur-6 Are Implicated in the Regulation of Exocyst-Mediated Membrane Trafficking in Caenorhabditis elegans
Source: G3 (Bethesda). 2013 Nov 5;4(1):173–83. doi: 10.1534/g3.113.006718 (PMC3887533; doi:10.1534/g3.113.006718)
Supplement: Supporting Information [file supp_g3.113.006718_TableS1.pdf]

Table S1 Oligo sequences for qRT-PCR

oligo name	oligo sequence
*pkc-3*_forward	5’ CCATCGGCATGTGCGAACGCA 3’
*pkc-3*_reverse	5’ TCCTCCACCCGACCGCGTTG 3’
*par-1*_forward	5’ GGAGCAACTGGTCCATCGGCCA 3’
*par-1*_reverse	5’ GCACGTCCTGATACTGGCTGGGT 3’
*sur-6*_forward	5’ CCTATCTGCCGACGATTTGCGAGT 3’
*sur-6*_reverse	5’ TGCGTTGGATGGAATTCGGCG 3’
*pptr-2*_forward	5’ CGTCGACCACGTTTCCGGAGC 3’
*pptr-2*_reverse	5’ TGCAGATGTGGCCAGGCAGC 3’
*rsa-1*_forward	5’ TGTTGGCGCCAGCGGACTTG 3’
*rsa-2*_reverse	5’ TGGCGGAGGCAGTGGTATGACG 3’
